# Combination Susceptibility Testing of Common Antimicrobials *in Vitro* and the Effects of Sub-MIC of Antimicrobials on *Staphylococcus aureus* Biofilm Formation

**DOI:** 10.3389/fmicb.2017.02125

**Published:** 2017-11-01

**Authors:** Bing Yang, Zhixin Lei, Yishuang Zhao, Saeed Ahmed, Chunqun Wang, Shishuo Zhang, Shulin Fu, Jiyue Cao, Yinsheng Qiu

**Affiliations:** ^1^Department of Veterinary Pharmacology, College of Veterinary Medicine, Huazhong Agricultural University, Wuhan, China; ^2^National Reference Laboratory of Veterinary Drug Residues and Key Laboratory for Detection of Veterinary Drug Residues, Huazhong Agricultural University, Wuhan, China; ^3^School of Animal Science and Nutritional Engineering, Wuhan Polytechinic University, Wuhan, China

**Keywords:** antimicrobial, sub-MIC, morphological observation, biofilm formation

## Abstract

The current study was conducted to evaluate the antibacterial combination efficacies, and whether the sub-inhibitory concentrations (sub-MIC) of antibiotics can influent on the biofilm formation of *S. aureus*. The minimum inhibitory concentration (MIC) of common antibacterial drugs was determined *in vitro* against clinical isolates of *Staphylococcus aureus* (*S. aureus*), *Escherichia coli* (*E. coli*), and *Pasteurella multocida* (*P. multocida*) alone and in combination with each other by using the broth microdilution method and the checkerboard micro-dilution method analyzed with the fractional inhibitory concentration index (FICI), respectively. Regarding these results, antibacterial drug combinations were categorized as synergistic, interacting, antagonistic and indifferent, and most of the results were consistent with the previous reports. Additionally, the effects of sub-MIC of seven antimicrobials (kanamycin, acetylisovaleryltylosin tartrate, enrofloxacin, lincomycin, colistin sulfate, berberine, and clarithromycin) on *S. aureus* biofilm formation were determined *via* crystal violet staining, scanning electron microscopy (SEM) and real-time PCR. Our results demonstrate that all antibiotics, except acetylisovaleryltylosin tartrate, effectively reduced the *S. aureus* biofilm formation. In addition, real-time reverse transcriptase PCR was used to analyze the relative expression levels of *S. aureus* biofilm-related genes such as *sarA, fnbA, rbf*, *lrgA, cidA*, and *eno* after the treatment at sub-MIC with all of the six antimicrobials. All antibiotics significantly inhibited the expression of these biofilm-related genes except for acetylisovaleryltylosin tartrate, which efficiently up-regulated these transcripts. These results provide the theoretical parameters for the selection of effective antimicrobial combinations in clinical therapy and demonstrate how to correctly use antibiotics at sub-MIC as preventive drugs.

## Introduction

In recent years, there has been an increasing tendency regarding the misuse of antimicrobials in clinical veterinary treatments. The widespread abuse of antibacterial drugs for the treatment of bacterial infections has led to the emergence and spread of drug-resistant. Presently, treatment with antimicrobials alone is not guaranteed to cure the serious and mixed infections. There is increasing clinical evidence on the base of combination therapy of antimicrobials are a valuable treatment option against the mixed or multidrug-resistant bacterial infections (Bharat et al., 2016; Stein et al., 2016; Wei et al., 2016). Combinations of antibacterial agents are a reasonable way to enhance the antibacterial action of drugs, especially for serious and mixed infections. *In vitro*, the activities of drugs combination can be determined by a checkerboard micro-dilution method or time-kill experiments with static antibiotic concentrations, which are usually used to evaluate susceptibility to combinations of antibiotics (Hall et al., 1983). According to standard definitions, the effects of antibiotic combinations are divided into synergy, interaction, indifference and antagonism. Many previous studies of antibiotic combinations have demonstrated that the antimicrobial effects of beta-lactams in combination with macrolides are antagonistic, but the reports are conflicting (Yoshioka et al., 2016; Lee et al., 2017). A study by Lodise *et al*. reported that beta-lactams were used in combination with macrolides in the treatment of community-acquired pneumonia (Lodise et al., 2007). In this study, the effects of common antibiotic combinations on *S. aureus, E. coli*, and *P. multocida*, representing both Gram-positive and Gram-negative bacteria, were investigated *in vitro*. In addition, quinolones, rifamycins, lincosamides and an extract of Chinese medicine were included as experimental drugs in this report.

Biofilms are a matrix composed of polymeric substances, DNA, and proteins that form a protective layer around bacteria (Donlan, 2002; Cue et al., 2009; Lee et al., 2014). Previous studies had been reported that biofilms can reduce the susceptibility of bacteria to antimicrobials (Stewart and Franklin, 2008). The development of bacterial biofilms can provide an extracellular barrier against the antimicrobial agents (Hsu et al., 2011). A review published by Ranita Roy *et al* has been reported the mechanism of *S. aureus* biofilm formation with the reference to different models and various methods used for anti-biofilm formation. In addition, various anti-biofilm molecules and their action mechanisms which may include herbal active compounds, chelating agents, peptide antibiotics, lantibiotics, and synthetic chemical compounds have been listed and introduced in this review (Roy et al., 2017). Some studies have demonstrated the antimicrobial agents at concentrations below their minimum inhibitory concentration (sub-MIC) have a clear influence on the biofilm formation (Majtán et al., 2008; Dong et al., 2012; El Haj et al., 2016). Some researchers have indicated that antimicrobial agents may inhibit or induce the bacterial biofilm formation at sub-MIC to avoid the chronic infection. For instance, Vishvanath Tiwari et al. have discovered and reported that the *Actinidia deliciosa* extract can reduce the production of exopolysaccharides (EPS), proteins and extracellular-DNA (e-DNA) to interference *Acinetobacter baumannii* biofilm formation (Tiwari et al., 2017). In addition, the sub-MIC of antibiotics could result in morphological changes and changes in the relative expression levels of biofilm-related genes in bacterial strains.

In order to determine the most effective drug combinations remedy against bacterial infections, evaluating the efficacy of drug combinations and discussing the effect of sub-MIC of drugs on biofilm formation, the following trails have been carried out. These investigations are based on the original classification of antibiotics, adding quinolones, rifamycins, lincosamides and herbal extracts-berberine, study their interactions between drugs on clinical isolates of *S. aureus, E. coli*, and *P. multocida*, representing both Gram-positive and Gram-negative bacteria, with representative drugs including amoxicillin, ceftiofur, kanamycin, colistin sulfate, doxycycline, acetylisovaleryltylosin tartrate, florfenicol, sulfadimidine, enrofloxacin, rifampicin, berberine, and lincomycin. In addition, we studied the effects of sub-MIC antibiotics on clinical bacteria biofilm formation. Bacteria were screened for their ability to form biofilms under experimental conditions and isolates were used to study the effect of sub-MIC of drugs on biofilm formation *via* crystal violet staining, scanning electron microscopy (SEM) and real-time polymerase chain reaction (RT-PCR). These experiments provide theoretical knowledge for veterinary clinical use and define the optimal antimicrobial combinations. We also warn people that the use of sub-MIC for the biofilm formation must be based on a laboratory research results.

## Materials and methods

### Bacterial strains

Clinically isolated strains of *S. aureus* (*S. aureus* Hb0206 isolated from pigs in Hubei, China), *E. coli* (*E. coli* Hd311 isolated from pigs in Hubei, China) and *P. multocida* (*P. multocida* 0625 isolated from pigs in Hubei, China) are provided by the microbiological lab of Huazhong Agriculture University. *Escherichia coli* ATCC_25922_ and *Staphylococcus aureus* ATCC_25923_ were chosen as quality control strains in all of the agent's susceptibility testing.

*Escherichia coli* and *Staphylococcus aureus* strains were cultured in 5 mL of nutrient broth at 37°C overnight. Then the *E. coli* strains were streaked from nutrient broth and grown on MacConkey agar plates at 37°C overnight. *S. aureus* strains were grown on nutrient agar plates at 37°C overnight. *P. multocida* strains were grown on TSA plate medium with 5% fetal bovine serum and were cultured at 37°C overnight. All of the incubation above aims to separate and purify isolated strains. A single bacterial colony was used to inoculate 5 mL Mueller-Hinton (MH) broth and was incubated at 37°C overnight, and *P. multocida* strains should with 5% fetal bovine serum.

### Preparation of antimicrobial agents

Kanamycin, doxycycline, acetylisovaleryltylosin tartrate, enrofloxacin, lincomycin, and colistin sulfate standards were obtained from the Wuhan Huisheng Biotechnology Group. Amoxicillin, ceftiofur, and rifampicin standards were purchased from Changfengda Commercial Trading Company (Wuhan, China). Florfenicol and sulfadimidine standards were purchased from the Biotechnology Research Institute (Beijing, China). The berberine standard was provided by the National Institutes for Food and Drug Control. Antibiotic stock solutions were prepared in advance and stored at −20°C. The concentration of all stock solutions was 5120 μg/mL.

### Combination susceptibility testing

Firstly, the MICs of each of the 12 drugs were determined in the three bacterial species by the broth microdilution method according to the Clinical and Laboratory Standards Institute (CLSI) M31-A3 guidelines (2012) (CLSI, 2008). The checkerboard micro-dilution method was used to test combination susceptibility of the 12 candidate antimicrobials in the three bacterial strains according to Pankey et al (Pankey and Ashcraft, 2009). Serial two-fold dilutions were prepared in broth ranging from 1/4 to 16-fold the MIC concentration for drug A and drug B, respectively. A total of 100 μL of 8 to 1/4-fold MIC concentration of drug A was added to row 1 and columns A-F, and then 50 μL of 16 to 1/2-fold MIC concentration of drug A was added to rows A-G and columns 2–7. A total of 100 μL of 1/4 to 8-fold MIC concentration of drug B was added to row H and columns 2–7, and then 50 μL of 1/2 to 16-fold MIC concentration of drug B was added to columns 2–7 and rows A-G. A total of 200 μL broth was added to column 1 and rows H. And the checkerboard plates were inoculated with 10^6^ c.f.u./mL of strains and incubated at 37°C for 24 h.

The FICI was calculated for each antimicrobial in each combination to evaluate the effect of the combination. The formulas used to calculate the FICI are as follows:

FICI of drug A = (MIC of drug A in combination) / (MIC of drug A alone)
FICI of drug B = (MIC of drug B in combination) / (MIC of drug B alone)
∑FICI = FICI of drug A + FICI of drug B

A stringent definition was used to explain the effect of combination susceptibility of the drugs. Synergy was defined as ∑FICI ≤ 0.5. The interaction was defined as 0.5 < ∑FICI ≤ 1. Indifference was defined as 1 < ∑FICI ≤ 2. Antagonism was defined as ∑FICI > 2. All tests were repeated in triplicate.

### Selection of bacteria with a strong ability to form biofilms

The biofilm formation assays were performed by the crystal violet staining method using sterile 96-well polystyrene plates. *Staphylococcus aureus, Escherichia coli*, and *Pasteurella multocida* were used in the experiments. Bacterial strains were used to inoculate 5 mL of TSB after an overnight culture on TSA at 37°C. The bacterial suspension was transferred to a 96-well polystyrene plate (100 μL/well) at a concentration of 1.0 × 10^7^ CFU/mL. Media lacking bacteria was used as a negative control.

After a 24 h incubation period at 37°C without shaking, the content of each well was removed, and the wells were carefully washed two times with sterile distilled water. The plates were air-dried in a ventilator and stained with 120 μL of a 1% w/v crystal violet solution. Crystal violet was removed after 15~20 min and the wells were washed with 300 μL of sterile distilled water to remove excess bacterial strains. The dye bound to the bacteria was dissolved thoroughly with 150 μL 33% glacial acetic acid for 10 min. The absorbance in each well was measured with a spectrophotometer at 630 nm (OD_630_).

The strains were classified into four different categories according to their biofilm-forming ability depending on their 2 OD_c_ (the average OD of the blank well at threefold its standard deviation) of bacteria. The categories were delineated as follows: not able to form a biofilm (OD_c_ < OD < 2 OD_c_), weakly able to form a biofilm (2 OD_c_ < OD < 4 OD_c_), moderately able to form a biofilm (4 OD_c_ < OD < 6 OD_c_), and strongly able to form a biofilm (6 OD_c_ < OD).

### Screening for optimum conditions of biofilm formation

*Staphylococcus aureus* was grown at 37°C in TSB as in previous studies. Strains that had a strong ability to form biofilms were cultured in TSB media at 37°C for 1–5 days. Subsequently, crystal violet staining was used to optically screen for the best conditions for bacterial biofilm formation.

### Effect of sub-MIC on biofilm formation with crystal violet staining

A strongly biofilm-forming isolate was selected to test the influence of sub-MIC of some antimicrobials on biofilm formation. The trail was performed in sterile 96-well polystyrene plates. The methods were the same as the selection test for identifying strong biofilm-forming isolates. The tested drugs were kanamycin, colistin sulfate, acetylisovaleryltylosin tartrate, enrofloxacin, berberine, lincomycin, and clarithromycin. The isolates were exposed to 1/2 MIC, 1/4 MIC, 1/8 MIC, 1/16 MIC, 1/32 MIC, 1/64 MIC and 1/128 MIC supplemented in TSB at 37°C for 48 h. Furthermore, wells lacking drugs and lacking bacteria in the same media served as the negative control and blank control, respectively. After incubation, biofilm formation was quantified by crystal violet staining. The tests were performed three times.

### Morphological observations by SEM

SEM was used to observe morphological variations in biofilm formation following treatment with antimicrobial agents. TSB was mixed with each antimicrobial agent at 1/2 MIC and was pipetted onto sterile culture dishes containing *S. aureus*. The same medium and inoculum, lacking agents, was used as a control. A sterile plastic coverslip was inserted into every dish. After overnight incubation at 37°C, the coverslips were carefully washed twice with sterile PBS following guidelines of the microscopy core laboratory at Huazhong Agricultural University. *S. aureus* biofilm formation on the coverslips was observed with a HITACHI SU8010.

### Real-time polymerase chain reaction (RT-PCR)

RT-PCR was used to analyze the relative expression of biofilm-related genes such as *sarA, fnbA, rbf*, *lrgA, cidA*, and *eno*. The primer sequences are listed in Table 1. Overnight cultures of *S. aureus* were added to fresh TSB and grown at 37°C with shaking. The drugs kanamycin, lincomycin, enrofloxacin, colistin sulfate, acetylisovaleryltylosin tartrate and berberine were dissolved in TSB to 1/2 MIC, 1/4 MIC, 1/8 MIC, 1/16 MIC, and 1/32 MIC. The mixture of TSB with antimicrobial agents at sub-MIC and *S. aureus* cultures were transferred into test tubes. Tubes lacking antibiotics were used as controls. All experimental and control tubes were incubated at 37°C overnight, then *S. aureus* cells were resuspended in lysostaphin (Sigma-Aldrich) and incubated at 37°C for 30 min. Then total RNA was extracted by the TRIzol (Takara) method followed by RNase-free DNase (Takara) treatment to eliminate any remaining DNA. The levels of mRNA were determined by real-time quantitative reverse-transcription PCR with the Quant Script RT Kit (Takara) and a Real Master Mix (SYBR Green II) Kit. All samples were carried out in triplicate and three independent experiments were performed. Expression levels of the genes were normalized to 16S rRNA. The changes in each transcript were determined by the 2^−ΔΔT^ method when compared to drug-free cells.

**Table 1:** Primers and sequences used in this study.

**Gene**	**Primer**	**Sequence (5′ → 3′)**	**References**	**Accession**
*sarA*	*sarA*-F	TCTTGTTAATGCACAACAACGTAA	Zielinska et al., 2012; Abdelhady et al., 2014	DQ414117.1
	*sarA*-R	TGTTTGCTTCAGTGATTCGTTT		
*fnbA*	*fnbA*-F	GAAGAGCATGGTCAAGCACA	Lee et al., 2014	KP096552
	*fnbA*-R	ACGTCATAATTCCCGTGACC		
*rbf*	*rbf*-F	ACGCGTTGCCAAGATGGCATAGTCTT	Cue et al., 2009	KR001974.1
	*rbf*-R	AGCCTAATTCCGCAAACCAATCGCT		
*lrgA*	*lrgA*-F	AGACGCATCAAAACCAGCACACT	Hsu et al., 2011	U52961.1
	*lrgA*-R	CCGGCTGGTACGAAGAGTAAGCC		
*cidA*	*cidA*-F	AGCGTAATTTCGGAAGCAACATCCA	Hsu et al., 2011	AY58192.1
	*cidA*-R	CCCTTAGCCGGCAGTATTGTTGGTC		
*eno*	*eno*-F	TGCCGTAGGTGACGAAGGTGGTT	Atshan et al., 2013	KU295448.1
	*eno*-R	GCACCGTGTTCGCCTTCGAACT		
*16Sr*	*16SrRNA*-F	GAGGGTCATTGGAAACTGGA	Lee et al., 2014	AH013003.2
*RNA*	*16SrRNA*-R	CATTTCACCGCTACACATGG		

### Ethics statement

All bacterium experiments and experimental protocols used in this study were conducted in accordance with the Guide for the Care and Use of Laboratory Animals of Hubei Provincial Laboratory Animal Service Center (permit number SYXK 2013-0044) and approved by the Ethics Committee of Huazhong Agricultural University, Wuhan, China.

### Statistical analysis

The experimental data were analyzed using a one-way analysis of variance (ANOVA) by SPSS 22.0 and the statistical results are reported as a mean ± standard deviation. Pairwise comparisons with differences of *P* ≤ 0.05 were considered statistically significant; *P* ≤ 0.01 was considered extremely significant.

## Results

### Combination susceptibility testing of 12 kinds of drugs

Firstly, the MICs of 12 different drugs in three species of bacteria were determined according to the CLSI standard and within the scope of quality control. The results are shown in Table 2.

**Table 2:** MICs of 12 different drugs in three species of bacteria.

**Formulation**	**MIC (**μ**g/mL)**		
	***S. ureus***	***E. coli***	***P. multocida***
Amoxicillin	1/8	32	1/2
Ceftiofur	2	1/8	1/16
Kanamycin	8	2	16
Colistin Sulfate	1	1/8	1
Doxycycline	32	32	1
A-Tartrate	2	128	128
Florfenicol	64	2	1
Sulfadimidine	8	16	16
Enrofloxacin	0.5	16	16
Rifampicin	8	4	1/2
Berberine	128	512	32
Lincomycin	1	512	512

*A-Tartrate stands for acetylisovaleryltylosin tartrate*.

Results showed that the strain of *S. aureus* was sensitive to most of the used drugs and resistant to doxycycline, florfenicol and berberine. *Escherichia coli* was susceptible to ceftiofur, kanamycin, colistin sulfate, florfenicol and rifampicin while resistant to amoxicillin, doxycycline, acetylisovaleryltylosin tartrate, sulfadimidine, enrofloxacin, berberine, and lincomycin. *P. multocida* was susceptible to amoxicillin, ceftiofur, colistin sulfate, doxycycline, florfenicol and rifampicin while resistant to kanamycin, acetylisovaleryltylosin tartrate, sulfadimidine, enrofloxacin, berberine and lincomycin.

The combination susceptibility of these 12 antibiotics on the three strains was assigned synergistic, interacting, antagonistic and indifferent according to the FICI.

The effects of antibacterial combinations on *S. aureus*, our representative Gram-positive bacteria, are shown in Table 3A. Enrofloxacin in combination with ceftiofur showed an additive effect (0.5 < FICI < 1), as well as rifampin in combination with ceftiofur or doxycycline and berberine in combination with florfenicol or sulfadimidine. When enrofloxacin was combined with doxycycline, florfenicol or sulfadimidine, when rifampin was combined with kanamycin or florfenicol, or when lincomycin was combined with acetylisovaleryltylosin tartrate or colistin sulfate, we observed an antagonistic effect (FICI > 2). The rest of the combinations had an indifferent effect (1 < FICI < 2). The FICI of berberine in combination with acetylisovaleryltylosin tartrate or ceftiofur was 1.5, indicating that these combinations could be applied to reduce toxicity and economize costs with fewer doses. In the current study, the doxycycline, florfenicol and berberine showed high MIC values against *S. aureus*, which indicates that *S. aureus* strains are resistant to these agents. Doxycycline in combining with berberine and florfenicol displayed the indifferent effects, while the berberine in combination with florfenicol showed an interactive effect. The synergistic effect was observed for the combination of acetylisovaleryltylosin tartrate and colistin sulfate against *S. aureus*, while sulfadimidine in combination with enrofloxacin showed an antagonistic effect against *S. aureus*. Notably, the isolate of *S. aureus* used in present study is sensitive to acetylisovaleryltylosin tartrate, colistin sulfate, sulfadimidine and enrofloxacin.

**Table 3A:** The FICI of 12 kinds of antibiotic against *S. aureus*.

**Agent A and B in combination**	**Effect on *S. aureus***	**Agent A and B in combination**	**Effect on *S. aureus***
amoxicillin+ceftiofur	Interaction	colistin sulfate+doxycycline	Synergy
amoxicillin+kanamycin	Interaction	colistin sulfate+florfenicol	Interaction
amoxicillin+colistin sulfate	Interaction	colistin sulfate+sulfadimidine	Interaction
ceftiofur+kanamycin	Interaction	doxycycline+rifampine	Interaction
ceftiofur+colistin sulfate	Interaction	A-Tartrate +florfenicol	Synergy
ceftiofur+sulfadimidine	Interaction	A-Tartrate +sulfadimidine	Synergy
ceftiofur+enrofloxacin	Interaction	florfenicol+sulfadimidine	Interaction
ceftiofur+rifampine	Interaction	florfenicol+Berberine	Interaction
kanamycin+colistin sulfate	Interaction	sulfadimidine+berberine	Interaction
kanamycin+sulfadimidine	Interaction		

The effects of antibacterial combinations on *P. multocida* (Table 3B) and *E. coli* (Table 3C), representative Gram-negative bacteria, are shown as follows. The FICI was 1.5, which suggested that they can be used together with fewer doses. Enrofloxacin in combination with ceftiofur, colistin sulfate, sulfamethazine or berberine showed a synergistic and additive effect, while enrofloxacin with florfenicol or rifampicin exhibited an antagonistic and indifferent effect. Rifampicin in combination with colistin sulfate, doxycycline, florfenicol or berberine showed an additive effect, while rifampicin in combination with kanamycin, lincomycin or enrofloxacin showed an antagonistic and indifferent effect. Berberine with amoxicillin or florfenicol had an indifferent effect, while all others showed an additive and synergistic effect. Lincomycin with colistin sulfate or sulfamethazine showed a synergistic and additive effect, while lincomycin with amoxicillin, kanamycin, acetylisovaleryltylosin tartrate, florfenicol or rifampin exhibited an antagonistic effect. The rest of the combinations had an indifferent effect. Besides, the combination of berberine and doxycycline resulted in an interactive effect *in vitro* against *E. coli* that is resistant to the individual drugs. Similarly, the combination of acetylisovaleryltylosin tartrate and berberine showed an interactive effect against *P. multocida* that is resistant to the individual drugs. The synergistic effect was observed when ceftiofur was combined with colistin sulfate and the antagonistic effect has been observed when ceftiofur was combined with florfenicol against *E. coli* which is sensitive to all individual drugs. Amoxicillin in combination with ceftiofur showed a synergistic effect against *P. multocida* that is resistant to the individual drugs. In contrast, the combination of amoxicillin and doxycycline showed an antagonistic effect against *P. multocida* that is resistant to doxycycline and amoxicillin.

**Table 3B:** The FICI of 12 kinds of antibiotic against *P. multocida*.

**Agent A and B in combination**	**Effect on *P. multocida***	**Agent A and B in combination**	**Effect on *P. multocida***
amoxicillin+ceftiofur	Synergy	colistin sulfate+rifampine	Interaction
amoxicillin+kanamycin	Interaction	colistin sulfate+berberine	Synergy
amoxicillin+colistin sulfate	Interaction	doxycycline+ A-Tartrate	Synergy
ceftiofur+kanamycin	Synergy	doxycycline+sulfadimidine	Interaction
ceftiofur+colistin sulfate	Interaction	doxycycline+rifampine	Interaction
ceftiofur+sulfadimidine	Interaction	doxycycline+berberine	Interaction
ceftiofur+enrofloxacin	Interaction	A-Tartrate+florfenicol	Interaction
ceftiofur+berberine	Interaction	A-Tartrate +berberine	Synergy
kanamycin+colistin sulfate	Interaction	florfenicol+sulfadimidine	Interaction
kanamycin+doxycycline	Interaction	sulfadimidine+enrofloxacin	Interaction
kanamycin+florfenicol	Interaction	sulfadimidine+berberine	Interaction
colistin sulfate+doxycycline	Interaction	enrofloxacine+berberine	Interaction
colistin sulfate+florfenicol	Interaction	rifampine+berberine	Interaction
colistin sulfate+sulfadimidine	Interaction		

**Table 3C:** The FICI of 12 kinds of antibiotic against *E. coli*.

**Agent A and B in combination**	**Effect on *E. coli***	**Agent A and B in combination**	**Effect on *E. coli***
amoxicillin+ceftiofur	Interaction	colistin sulfate+berberine	Interaction
amoxicillin+kanamycin	Synergy	colistin sulfate+lincomycin	Synergy
amoxicillin+colistin sulfate	Interaction	doxycycline+A-Tartrate	Interaction
ceftiofur+kanamycin	Synergy	doxycycline+berberine	Interaction
ceftiofur+berberine	Synergy	A-Tartrate+florfenicol	Interaction
kanamycin+colistin sulfate	Interaction	A-Tartrate+sulfadimidine	Interaction
kanamycin+sulfadimidine	Interaction	A-Tartrate +berberine	Interaction
kanamycin+berberine	Interaction	florfenicol+rifampine	Interaction
colistin sulfate+doxycycline	Interaction	sulfadimidine+enrofloxacin	Interaction
colistin sulfate+doxycycline	Synergy	sulfadimidine+berberine	Interaction
colistin sulfate+sulfadimidine	Interaction	sulfadimidine+lincomycin	Interaction
colistin sulfate+enrofloxacin	Synergy	enrofloxacine+berberine	Interaction
colistin sulfate+rifampine	Interaction	rifampine+berberine	Interaction

*The results showed in Table 3 are drug-combinations had synergy and interaction. The rest of the combinations had indifferent or antagonistic effects. A-Tartrate stands for acetylisovaleryltylosin tartrate*.

### Bacterial strains with a strong ability to form biofilms

Biofilm formation assays were performed using the crystal violet staining method. The results are shown in Table 4.

**Table 4:** OD_630_ of 3 isolates.

**Isolate**	**Control group**	***S. aureus***	***E. coli***	***Pasteurella***
OD_630_	0.052 ± 0.001	0.432 ± 0.01^**^	0.124 ± 0.004^*^	0.061 ± 0.001

*OD_630_ means the absorbance of biofilm was measured with a spectrophotometer at 630 nm*.

* *Represent statistical significance (p ≤ 0.05)*,

** *Represent extreme significance (p ≤ 0.01)*.

An isolate of *S. aureus* that had an OD_630_ greater than 6 OD_c_ was considered to be a strong biofilm-forming strain. Isolates of *E. coli* and *P. multocida* that had 2 OD_c_ < OD < 4 OD_c_ or OD_c_ < OD < 2 OD_c_ were considered to be weak biofilm-forming strains or unable to form biofilms, respectively. *S. aureus* was used as the experimental strain.

### Optimizing conditions for biofilm formation

Previous studies have demonstrated that stains of *S. aureus* grow well at 37°C in tryptic soy broth (TSB). The culture time was identified through experimental means. The results are shown in Table 5. There was evidently biofilm formation after 1 day of culture at 37°C, and biofilm formation reached its peak after 3 days of culture as determined by OD_630_ measurements. The difference between biofilm formation after 1, 4, and 5 days was not significant, and there was no significant difference between 2 and 3 days (*P* > 0.05). But there were significant differences between 1, 4, or 5 days and 2 or 3 days (*P* ≤ 0.05) Table 5. According to the experimental data and statistical analysis, the optimal experimental conditions for biofilm formation were to culture for 2 days in TSB at 37°C.

**Table 5:** Influence of time on *S. aureus* biofilm formation.

**Days**	**0**	**1**	**2**	**3**	**4**	**5**
OD_630_	0.052 ± 0.002	0.420 ± 0.03	0.619 ± 0.03^*^	0.625 ± 0.03^*^	0.550 ± 0.03	0.423 ± 0.02

*OD_630_ means the absorbance of biofilm was measured with a spectrophotometer at 630 nm*.

* *Represent statistical significance (p ≤ 0.05)*.

### Effect of sub-MIC on biofilm formation with crystal violet staining

**Figure 1:**
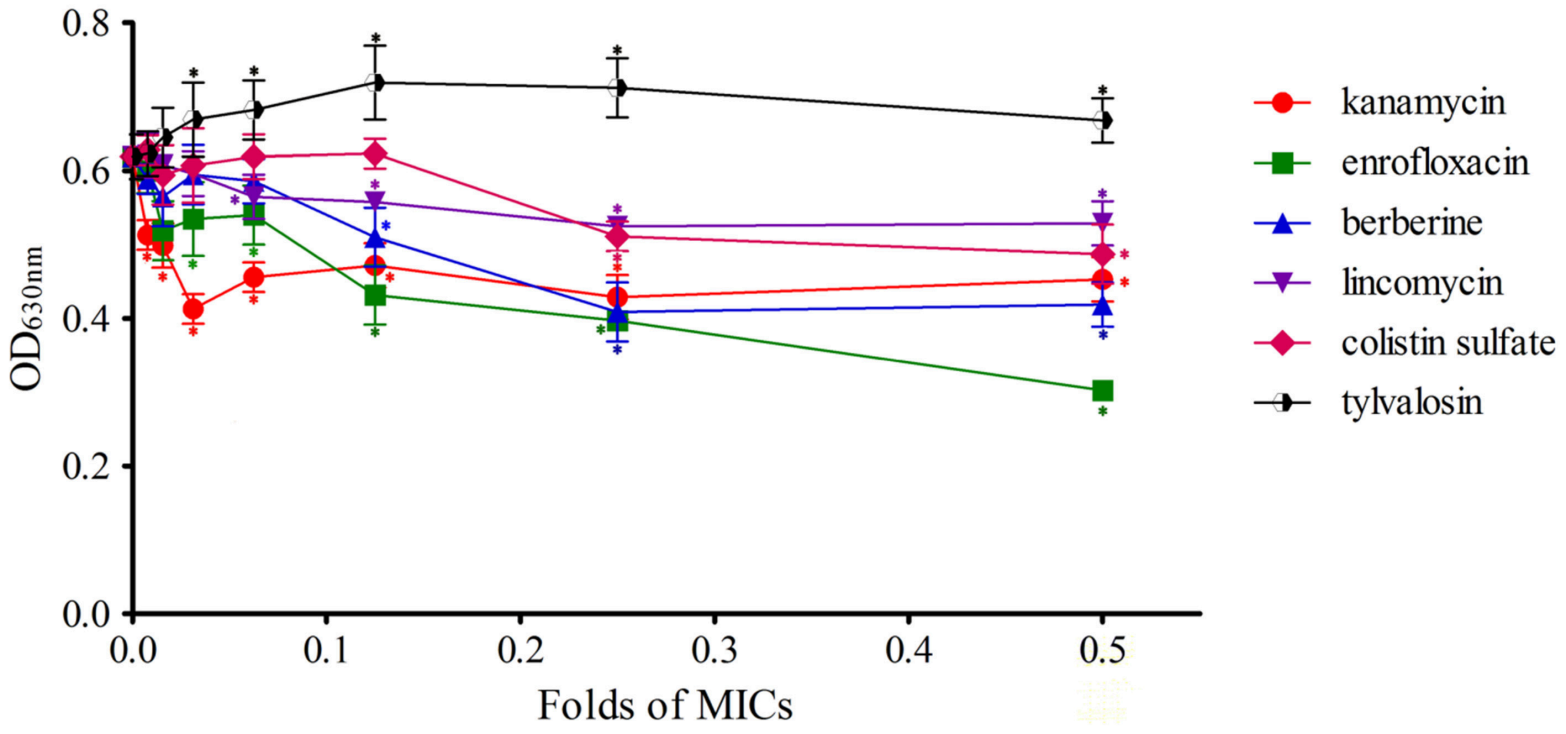
Effects of antimicrobial sub-MIC on *S. aureus* biofilm formation with crystal violet staining. The biofilms were stained with crystal violet and the optical density was determined with a multi-detection microplate reader at a wavelength of 630 nm (OD_630_). Data shown are representative three independent experiments and are expressed as mean ± SD. The treated isolates exhibit a significant reduction in biofilm formation for enrofloxacin, lincomycin, kanamycin, berberine, and colistin sulfate, but not for acetylisovaleryltylosin tartrate. ^*^Represent statistical significance (*p* ≤ 0.05).

The biofilm formation of *S. aureus* treated with sub-MIC of different kinds of drugs was determined using crystal violet staining. Values were expressed as means of OD_630_ ± standard deviations. Details of the results are shown in Figure 1.

*Staphylococcus aureus* biofilms incubated with kanamycin were significantly inhibited compared to the control (*P* ≤ 0.05), and the difference between each concentration tested was not significant. These results indicated that the different concentrations of kanamycin used are irregular in their ability to influence the inhibition of biofilm formation.

The levels of biofilm formation for *S. aureus* treated with 1/128 of the MIC of enrofloxacin exhibited no significant inhibition relative to the control (*P* > 0.05) but had a significant difference at other concentrations (1/64~1/2 MIC). Inhibition was positively correlated with the concentration of enrofloxacin at 1/16~1/2 MIC.

Biofilm formation of *S. aureus* during treatment with berberine was significantly inhibited at concentrations of 1/8~1/2 MIC compared to the control (*P* ≤ 0.05), and there was no significant difference between 1/128~1/16 MIC treatments and the drug-free group (*P* > 0.05). But the difference between 1/8 MIC treatment and 1/2 MIC treatment was not significant (*P* > 0.05).

Biofilm formation in *S. aureus* incubated with 1/16~1/2 MIC lincomycin was significantly inhibited relative to the drug-free group (*P* ≤ 0.05), and the inhibitory effects were not significant between groups. The difference between other concentrations and the control were not significant (*P* > 0.05), indicating that biofilm growth was not affected by these concentrations of lincomycin.

Colistin sulfate at 1/4 and 1/2 MIC had a significant inhibitory effect on biofilm formation compared to the control (*P* ≤ 0.05), and the difference among groups was not statistically significant. The difference between lower concentrations (1/128~1/8 MIC) of colistin sulfate and the drug-free group was not statistically significant (*P* > 0.05) at inhibiting biofilm formation.

Biofilm formation was observably enhanced at 1/32~1/2 MIC of acetylisovaleryltylosin tartrate, revealing that acetylisovaleryltylosin tartrate at these concentrations significantly induced biofilm formation relative to the control (*P* ≤ 0.05). The inducing effect of acetylisovaleryltylosin tartrate on biofilm formation was in agreement with changes at 1/32~1/4 MIC. But the inducing effect was not significantly different when compared to the drug-free group at 1/64 MIC (*P* > 0.05). Thus, a lower dose of the drug may be the primary factor responsible for the decrease in its inducing effect.

**Figure 2:**
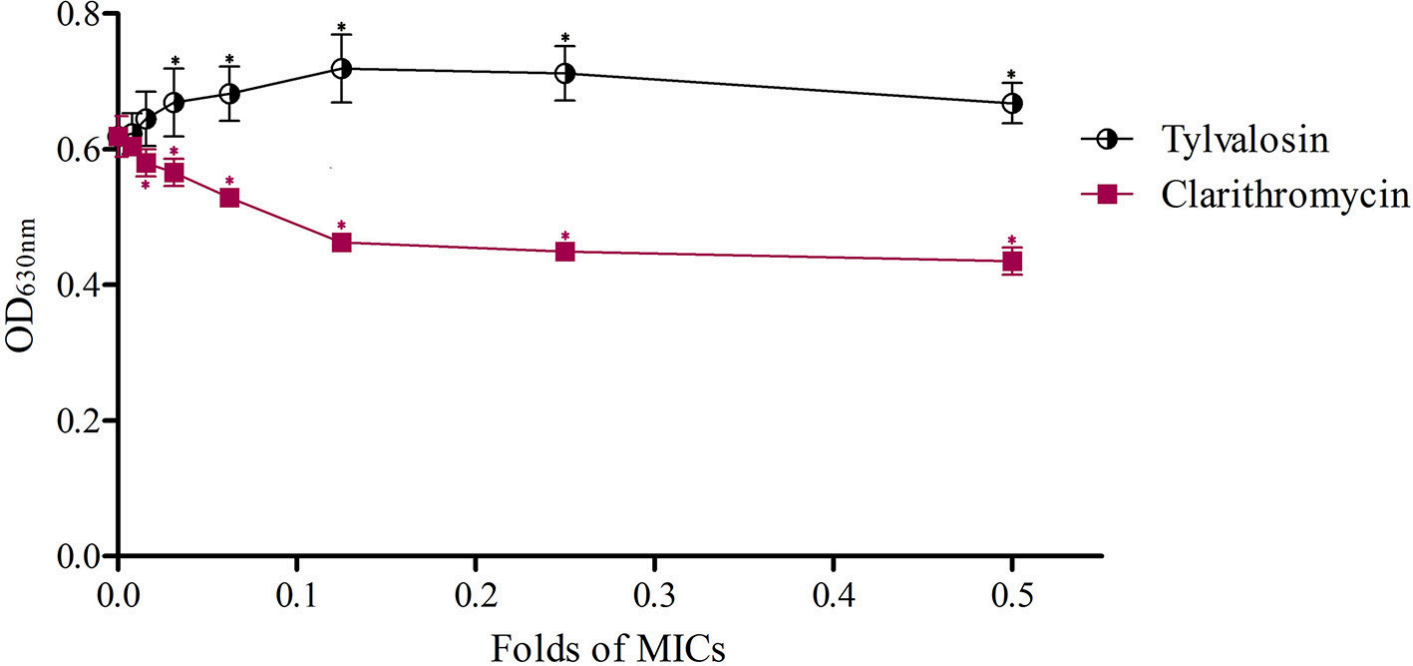
Effects of clarithromycin and acetylisovaleryltylosin tartrate at sub-MIC on *S. aureus* biofilm formation with crystal violet staining. The biofilms were stained with crystal violet and the optical density was determined with a multi-detection microplate reader at a wavelength of 630 nm (OD_630_). Data shown are representative three independent experiments and are expressed as mean ± SD. Sub-MIC of acetylisovaleryltylosin tartrate significantly induced *S. aureus* biofilm formation but clarithromycin inhibited biofilm formation compared with drug-free bacteria. ^*^Represent statistical significance (*p* ≤ 0.05).

To further confirm the effects of macrolides on *S. aureus* biofilm formation, we evaluated the action of clarithromycin on biofilm growth *in vitro*. The treated isolate exhibited a significant reduction (*P* ≤ 0.05) in biofilm formation for 1/64~1/2 MIC clarithromycin in comparison with an isolate of drugs-free incubation. And the biofilm formation treated with a lower dose of clarithromycin was not significantly inhibited compared with drug-free groups. The results are shown in Figure 2.

### Morphological observations by SEM

**Figure 3:**
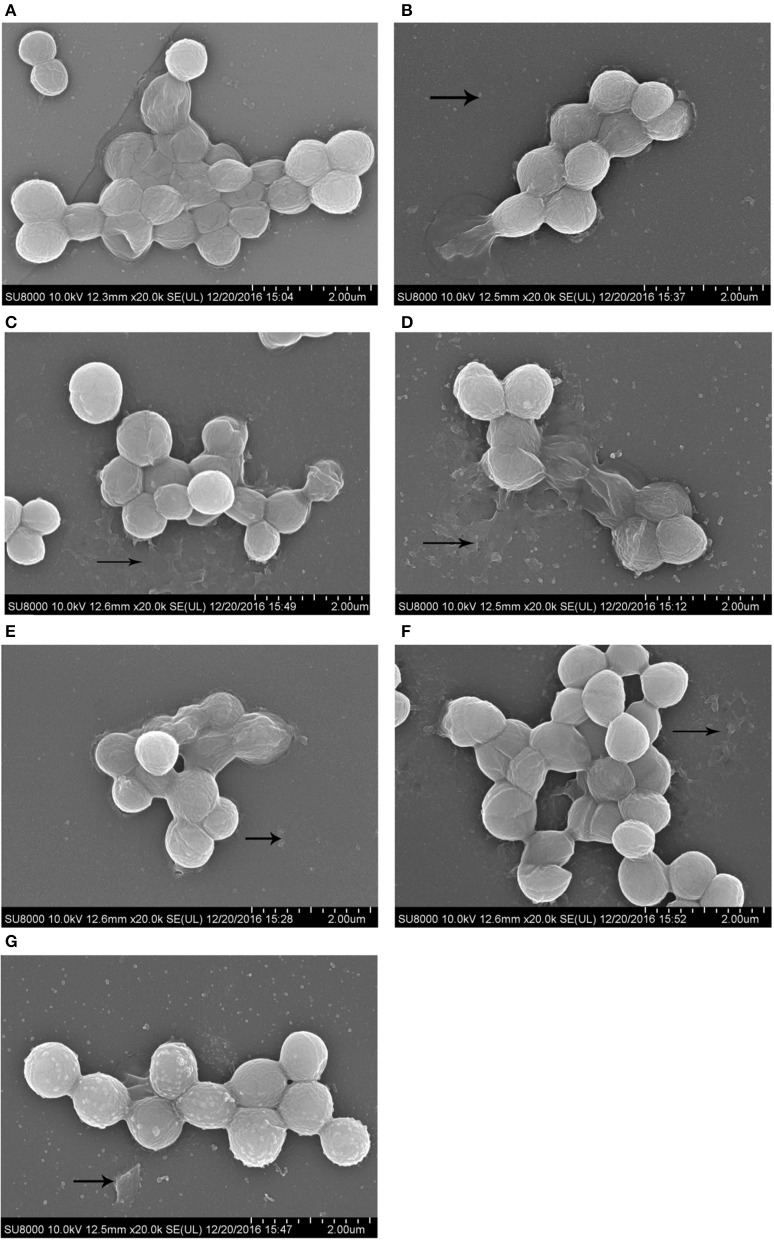
SEM images of biofilm formed by *S. aureus* incubated with 1/2 MIC of antibiotics for 72 h. Scale bars = 2.00 μm. **(A)**, drugs-free group; **(B)**, enrofloxacin treatment group; **(C)**, colistin sulfate treatment group; **(D)**, lincomycin treatment group; **(E)**, berberine treatment group; **(F)**, kanamycin treatment group; **(G)**, acetylisovaleryltylosin tartrate treatment group.

Morphology of the *S. aureus* biofilm on the surface of coverslips was observed using SEM at 2000X magnification. SEM results were consistent with those from our crystal violet staining observations. Images of biofilms formed by *S. aureus* in the presence of sub-MIC of antibiotics are shown in Figure 3.

### Real-time polymerase chain reaction (RT-PCR)

**Figure 4:**
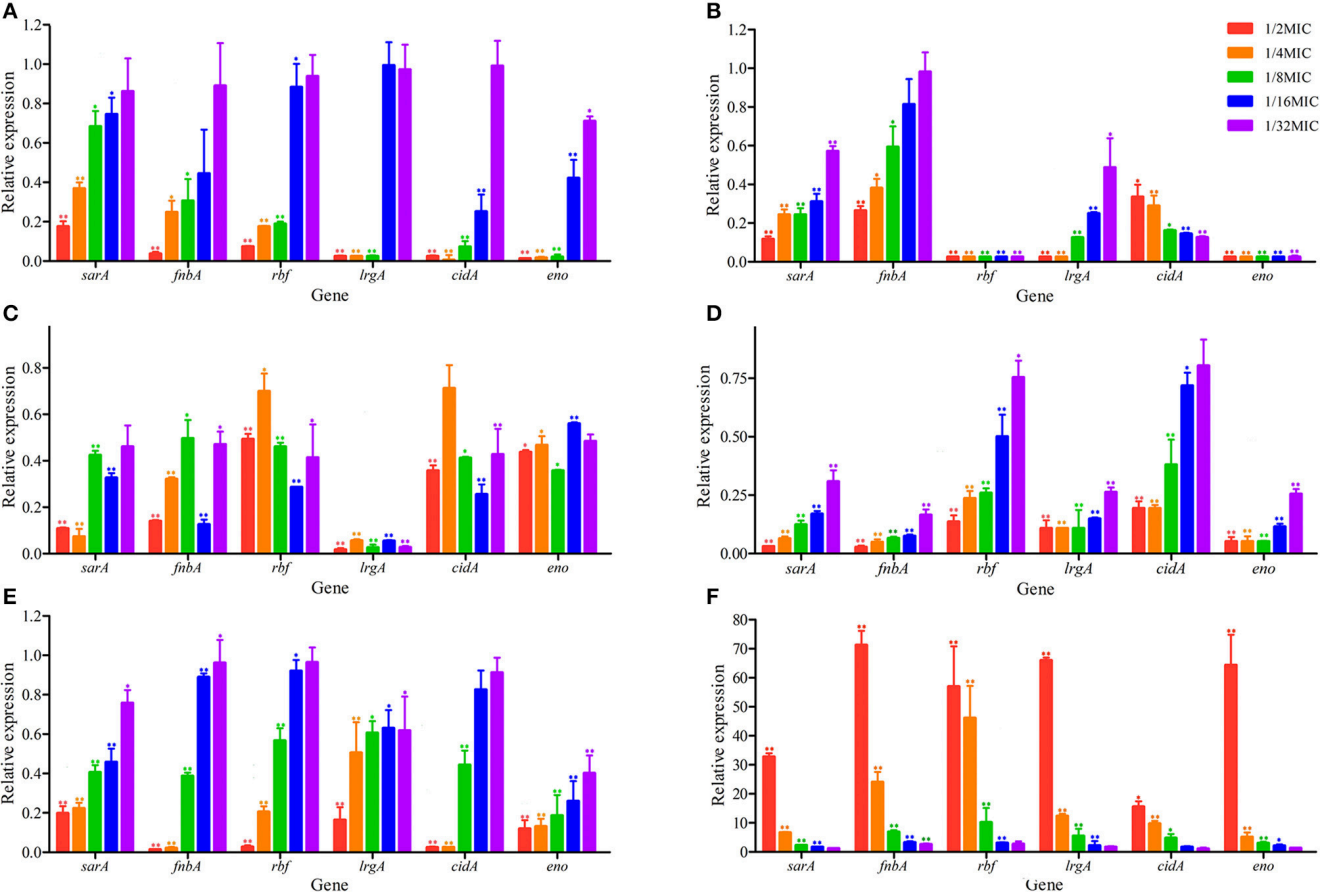
Effects of sub-MIC of antibiotics on *S. aureus* biofilm-related genes relative expression. **(A)**, berberine treatment groups; **(B)**, lincomycin treatment groups; **(C)**, kanamycin treatment groups; **(D)**, enrofloxacin treatment groups; **(E)**, colistin sulfate treatment groups; **(F)**, acetylisovaleryltylosin tartrate treatment groups. Expression levels of genes are expressed relative to that of 16SrRNA, which was assigned a value of 1. All data are shown as the Mean ± SD from three independent experiments. ^*^Represent statistical significance (*p* ≤ 0.05), ^**^Represent extreme significance (*p* ≤ 0.01).

To determine whether the influence of antibiotics was manifested at the transcriptional level, total RNA was isolated from *S. aureus* following treatment at sub-MIC with antibiotics. The transcript levels for all biofilm-related genes are shown in Figure 4. In *S. aureus* strains, the down-regulation of all genes was observed by RT-PCR at sub-MIC treatments of kanamycin, lincomycin, enrofloxacin, berberine, and colistin sulfate. But the acetylisovaleryltylosin tartrate treatment group showed up-regulation for all genes. RT-PCR results showed that the down-regulation of all biofilm-related genes following berberine treatment was significant at 1/8~1/2 MIC compared to the other groups (*P* ≤ 0.01; Figure 4A. The lincomycin treatment group exhibited a significant decrease in the relative expression levels of all genes, except for *fnbA*, following treatment with 1/32~1/16 MIC lincomycin (*P* ≤ 0.01; Figure 4B. The genes of *fnbA, rbf, lrgA*, and *cidA* were inhibited significantly following treatment with kanamycin at all sub-MIC, although the genes *sarA* and *eno* were not significantly different at 1/32 MIC (Figure 4C. Transcription levels for all genes were markedly decreased following treatment with enrofloxacin at sub-MIC (*P* ≤ 0.01), although the relative expression level of *cidA* was not significantly different at 1/32 MIC of enrofloxacin (Figure 4D). Similar to the enrofloxacin treatment group, the colistin sulfate treatment group showed significant inhibition in the relative expression levels of all genes except for *cidA* (Figure 4E). Except for *cidA*, the transcript levels for all genes in biofilms treated with acetylisovaleryltylosin tartrate at sub-MIC had increased significantly, and at 1/2 MIC were extremely elevated (Figure 4F). And to assess the effects of macrolides with different member-ring on *S. aureus* biofilm formation, an addition assay that clarithromycin influent on biofilm formation was investigated to compare with acetylisovaleryltylosin tartrate treatment group.

## Discussion

### Combination susceptibility testing of 12 kinds of drugs against three bacterial isolates

Existing knowledge about antibiotic combination therapy categorizes the effects of drug combinations as synergistic, interacting, indifferent and antagonistic. *In vitro*, the FICI obtained by the checkerboard dilution method can be usually used for the evaluation of antibiotic combinations (Pankey and Ashcraft, 2005). In the current study, a checkerboard micro-dilution method was used to analyze the antimicrobial activity of 12 kinds of drugs in alone and in combination with others against *S. aureus, E. coli*, and *P. multocida* to determine the value of combining conventional antibiotics. The results were consistent with previous studies.

At present, the effect of using antibiotics targeting the replication phase in combination with antibiotics targeting the stationary phase is controversial. Our results revealed that the combination of these two drugs types exhibited indifference and antagonism against *S. aureus*, and the most combinations exhibited the indifference and antagonism against *E. coli* and *P. multocida*. Macrolide antibiotics have a strong ability to disrupt the biofilms; thus macrolides are often used in conjunction with β-lactams or fluoroquinolones to enhance the ability of these antibiotic agents to treat biofilm-associated infections (Kandemir et al., 2005). Evidence indicates that fluoroquinolones used in combination with β-lactams have a clear synergistic effect against *Pseudomonas aeruginosa in vitro* (Chachanidze et al., 2009). Our study demonstrated some *in vitro* interaction and indifference when enrofloxacin was used with β-lactams against all of the tested bacterial strains. And enrofloxacin in combination with colistin sulfate, sulfadimidine and berberine had the effects of synergy or interaction and antagonism against Gram-negative and Gram-positive bacteria, respectively. For enrofloxacin, the mechanism of action was to antagonize the A subunit of the bacterial enzyme DNA gyrase and thus block the DNA replication (Wolfson and Hooper, 1985). And the bacteriostatic action of sulfanilamide is due to its interference with a hypothetical essential and anti-sulfanilamide metabolite of bacteria (Eyster, 1943). It was not surprising that enrofloxacin in combination with sulfadimidine showed a synergistic effect against *E. coli* and *P. multocida*. The combination of enrofloxacin with florfenicol against *S. aureus, E. coli*, and *P. multocida* led to antagonistic or indifferent effect, with doxycycline against *S. aureus* showed an interaction and with rifampin against *P. multocida* showed an antagonistic interaction. Rifampin specifically inhibits bacterial RNA polymerase by forming a stable drug-enzyme complex to reduce the efficacy of enrofloxacin (Wehrli, 1983). These results suggest that fluoroquinolones plus β-lactams, polymyxins, sulfadimidine, and berberine may be alternative combinations for severe infections. Now, rifampin is widely used in clinics and has been proved especially effective in the treatment of tuberculosis (Binda et al., 1971). In the current study, the combination of rifampin with other antimicrobials has been investigated. The combination of rifampin with ceftiofur had the effect of interaction against *S. aureus*, in contrast, which had the effect of indifference against *E. coli* and *P. multocida*. And rifampin in combination with doxycycline against *S. aureus* and *P. multocida* showed interactive effects. However, when rifampin was combined with kanamycin and lincomycin mainly antagonistic activities were observed. In addition, berberine, traditional medicine, was used to increase its effect on susceptibility when combined with common antibiotics. It has been reported that berberine has weak activity against Gram-negative bacteria and is more active against Gram-positive bacteria including *S. aureus* (Vuddanda et al., 2010). In our study, berberine combined with other drugs all exhibited synergy and interaction against Gram-negative bacteria, except for combinations with amoxicillin, florfenicol and lincomycin. Recently, berberine was demonstrated as a strong synergist for many common antibiotics against clinical bacteria (Yu et al., 2005; Zuo et al., 2012). However, the underlying mechanism of the synergistic action of berberine besides bactericidal activities should be a further study.

Regarding the combination susceptibility tests of the current study has been revealed that antibiotics combining against different bacterial strains displayed the different effects. For instance, the indifferent effect between acetylisovaleryltylosin tartrate and berberine was found against *S. aureus* which is sensitive to acetylisovaleryltylosin tartrate and resistant to berberine. the most synergistic effect has been observed for the combination of acetylisovaleryltylosin tartrate and berberine against *P. multocida*, which is all resistant to acetylisovaleryltylosin tartrate and berberine. Similarly, colistin sulfate combined with acetylisovaleryltylosin tartrate demonstrated a strong synergic effect against *S. aureus* and *E. coli in vitro* but showed an interactive effect against *P. multocida*. Importantly, the MIC values suggested that *E. coli* showed a highly resistant to acetylisovaleryltylosin tartrate than *S. aureus* strains, and the strains of *E. coli* and *P. multocida* are all resistant to acetylisovaleryltylosin tartrate. These data suggested that the combinations of antimicrobial agents that exhibit synergy or partial synergy may augment the antimicrobial effect in resistant strains at a lower concentration. In addition, the combined antimicrobial therapy is usually applied to expand the antimicrobial spectrum and reduce the inhibitory concentration of drug-resistant bacterium.

Current study clearly indicates that the antimicrobial combinations of enrofloxacin plus colistin sulfate or sulfadimidine, rifampin plus colistin sulfate, doxycycline or florfenicol, lincomycin plus colistin sulfate, or sulfadimidine could be used as a remedy for the serious infections caused by a Gram-positive bacterium such as *S. aureus*. In contrast, the combinations of enrofloxacin plus doxycycline, florfenicol or sulfadimidine, rifampin plus kanamycin or florfenicol and lincomycin plus colistin sulfate are not recommended for the treatment of Gram-positive bacterial infections. The combinations of enrofloxacin plus colistin sulfate or sulfadimidine, rifampin plus doxycycline, colistin sulfate or florfenicol, lincomycin plus colistin sulfate or sulfamide are suggested to be used for treating the Gram-negative bacterial infections, such as *E. coli* and *P. multocida*. Regarding current results, the combinations of berberine to most of the antibiotics in our tests were found to show a synergistic or interactive effect in addition to amoxicillin, florfenicol and lincomycin.

### Effects of sub-MIC of antibiotics on *S. aureus* biofilm formation

Biofilm is a matrix of self-produced extracellular polymeric substances such as exopolysaccharides, proteins, nucleic acids and other substances. And biofilms can play an important role in physical and biological properties such as slow growth or mechanical barrier and in the development of antimicrobial resistance. *Staphylococcus aureus* is usually used in biofilm-related trials as it is adept at forming biofilms (Hsu et al., 2011). And crystal violet staining is the most common assay used to qualify biofilms. Until now, there have been very few studies carried out on the effects of sub-MIC of antibiotics on biofilm formation (Hoffman et al., 2005; Dong et al., 2012; Henry-Stanley et al., 2014). While previous studies of the effects of antibiotics on biofilm formation have largely focused on one type of agent at 1/2 MIC, little is known about the use of several antibiotics at 1/128~1/2 MIC (Lee et al., 2014; Silva et al., 2014). In this study, a wide range of sub-MIC of antimicrobial agents was selected to detect biofilm formation. In our study, we investigated the *in vitro* effects of sub-MIC of aminoglycosides (kanamycin), fluoroquinolones (enrofloxacin), lincosamides (lincomycin), polypeptides (colistin sulfate), medicinal plant alkaloids (berberine) and macrolides (acetylisovaleryltylosin tartrate) on biofilm formation in *S. aureus* at 1/128~1/2 MIC. In addition to common antibiotics, we included the plant extract berberine to expand the scope of experimental agents. In addition, biofilm formation in *S. aureus* is the final result of the coordination of many different mechanisms as described in previous reports (Hsu et al., 2011). In this study, the genes *sarA, fnbA, rbf*, *lrgA, cidA*, and *eno* of *S. aureus* were selected to investigate transcription levels using RT-PCR following antibiotic treatment. Recent studies have demonstrated that all genes have a positive effect on *S. aureus* biofilm formation. *sarA* encodes multiple extracellular proteases, nucleases, and fibronection-binding proteins that play a role in *S. aureus* biofilm formation (Dunman et al., 2001; Cheung et al., 2008; Zielinska et al., 2012). *fnbA* (fibronectin-binding proteins) encodes proteins to mediate bacterial adherence (Hsu et al., 2011). *lrgA* and *cidA* encode antiholing-like and holing-like proteins, respectively (Rice et al., 2007). And *eno* encodes laminin-binding proteins (Atshan et al., 2013). The gene *rbf* is a transcriptional regulator of the *AraC* family (Cue et al., 2009). Our morphological observations and crystal violet staining revealed that biofilm formation following sub-MIC treatments of kanamycin, lincomycin, enrofloxacin, colistin sulfate, and berberine was significantly decreased relative to the negative control. But the sub-MIC in the acetylisovaleryltylosin tartrate treatment group had an obvious increase. Furthermore, our RT-PCR results revealed that the following antibacterial treatment, the expression levels of all genes were in agreement with changes in the biofilm.

Previous studies have been reported that aminoglycoside, fluoroquinolone and polypeptide antibiotics all efficiently prevent *S. aureus* biofilm formation (Saising et al., 2012; Ahire et al., 2013; Hess et al., 2013). The results of our study show that *S. aureus* biofilms incubated with kanamycin, enrofloxacin and colistin sulfate at sub-MIC were significantly inhibited as compared to the control. Hoffman et al. have shown that sub-MIC of aminoglycoside antibiotics induce biofilm formation in *P. aeruginosa* via contributing to aminoglycoside response regulator (*Arr*) (Hoffman et al., 2005). The results published by Wasfi et al. showed that ciprofloxacin, a fluoroquinolone antibiotic, was found to be the most effective in decreasing *S. aureus* and *Enterobacter sp*. biofilm formation (Wasfi et al., 2012). Wojnicz et al. also observed that the antibiotics of ciprofloxacin, amikacin, and colistin at sub-MIC all reduced *E. coli* biofilm formation *in vitro* and the inhibitory effects of sub-MIC of agents on biofilm formation was dependent on which antibiotics interfere with the expression of curli fimbriae (Wojnicz and Tichaczek-Goska, 2013).

Data from our study indicated that modest concentration of berberine and lincomycin (1/16-1/2 MIC) were sufficient to inhibit biofilm formation significantly. The lincosamides are known to interact with the 50S ribosomal subunit and affect translation by stimulating peptidyl-tRNA dissociation from the ribosomal particle (Menninger et al., 1994). A study by Huang showed that lincosamide antibiotics inhibited biofilm formation in *S. aureus* (Huang et al., 2012), as did lincomycin in our study. Berberine, a natural bioactive compound, has proven efficacy against microorganisms (Wojtyczka et al., 2014). Chu et al. (2016) have reported that berberine can inhibit MRSA biofilm formation via affecting amyloid fibril formation. Data from Wang et al. investigation indicated that modest concentrations of berberine (30–45 μg/mL) were sufficient to inhibit *Staphylococcus epidermidis* biofilm formation and the inhibitory effect may be related to the interaction of berberine and bacterial DNA to interfere with the transcription of target genes (Wang et al., 2009). However, the underlying mechanism by which traditional medicines inhibit biofilm formation and enhance antibacterial activity requires further study.

In our study, biofilm formation of *S. aureus* was dose-dependently induced by incubation with acetylisovaleryltylosin tartrate. Cirioni et al. found that clarithromycin, a macrolide antibiotic, can effectively prevent *P. aeruginosa* biofilm formation (Cirioni et al., 2011). However, studies by Aka et al. suggested that *P. aeruginosa* biofilm could be induced followed incubation in sub-MIC of azithromycin, a 15-membered macrolide antibiotic (Aka and Haji, 2015). Previous studies have proved that macrolide antibiotics such as 14, 15-membered can inhibit the production of alginate in mucoid strains and the activity of guanosine diphosphomannose dehydrogenase (GMD), the key enzyme in alginate synthesis is decreased, while the 16-membered macrolides have no effect on alginate production (Li et al., 2004). Clarithromycin, 14-membered macrolides, can inhibit the formation of *S. aureus* biofilm in our study. Moreover, clarithromycin has been shown to prevent biofilm formation by *S. aureus* (Dicicco et al., 2012). Although controversial, there is increasing evidence for the anti-biofilm properties of Staphylococcal biofilms *in vitro* (Jabalameli et al., 2012). This indicates that not all antibiotics have inhibited effects on *S. aureus* biofilm formation, even if these antibiotics belong to the same group. However, the underlying mechanisms of acetylisovaleryltylosin tartrate induce biofilm formation are poorly understood and need other investigation and exploration.

As described earlier, biofilm formation is highly associated with antibacterial stimulation. In this study, the effects of sub-MIC of antibiotics on *S. aureus* biofilm formation were explored from apparent to genetic. Crystal violet staining was used to investigate the influence of sub-MIC of antibacterial on biofilm formation. And the biofilm changes of *S. aureus* were further investigated using SEM. Consistent with crystal violet staining and morphological observations, the relative expression levels of biofilm-related genes, as determined by qRT-PCR, followed the same trends. Furthermore, the ratio of biofilm formation on a surface was consistent with changes in expression levels between different drug treatment groups. These outcomes suggested that biofilm formation depends on biofilm-related genes and associated proteins under treatment with sub-MIC of antibiotics. And similar results have been reported previously (Bai et al., 2015; Chen et al., 2016). Nevertheless, the mechanisms by which drugs affect biofilm formation should be investigated further.

## Conclusions

Our data showed synergistic effects of enrofloxacin plus ceftiofur, rifampin plus ceftiofur or doxycycline, berberine plus florfenicol or sulfadimidine against *S. aureus in vitro*. The findings suggest that these reasonable combinations could be used to remedy serious infections caused by Gram-positive bacteria. The antimicrobial effects of enrofloxacin in combination with ceftiofur, colistin sulfate, or sulfadimidine, rifampin in combination with colistin sulfate, doxycycline or florfenicol, lincomycin in combination with colistin sulfate or sulfadimidine on Gram-negative bacteria *in vitro* are showed addition, what seems to suggest these combinations could be used to remedy Gram-negative bacteria infectious disease. Our study addressed an important clinical concern that reasonable combination of antibiotics may enhance their antibacterial activity and better cure serious and mixed infections.

Results of biofilm formation assays suggest that sub-MICs of enrofloxacin, lincomycin, kanamycin, colistin sulfate, berberine, and clarithromycin inhibit *S. aureus* biofilm formation. And the biofilm formation was dose-dependently inhibited by incubation with berberine and lincomycin, but not enrofloxacin, kanamycin or colistin sulfate, which suggest that sub-MIC of drugs should be selected correct concentrations. However, the sub-MICs of acetylisovaleryltylosin tartrate induce the ability of biofilm formation of *S. aureus*, which indicated that antibiotics belong to the same groups with strains could present different effect on biofilm formation, for instance, sub-MIC of acetylisovaleryltylosin tartrate induced *S. aureus* biofilm formation but clarithromycin inhibits biofilm formation.

In conclusion, our study offers information on how to select the right antibacterial combinations for the therapy and how to correctly administer the drugs at sub-MIC for preventive use.

## Author contributions

JC conceived and designed the experiments. BY and YZ was responsible for performing the experiments, collecting and analyzing data, and writing the manuscript. ZL, BY, and CW contributed to experiments design, data processing, and experiment performing. SA, SZ, SF, and YQ participated in revised the manuscript. All authors read and approved the final manuscript.

### Conflict of interest statement

The authors declare that the research was conducted in the absence of any commercial or financial relationships that could be construed as a potential conflict of interest.
